# An Automated Recording Method in Clinical Consultation to Rate the Limp in Lower Limb Osteoarthritis

**DOI:** 10.1371/journal.pone.0164975

**Published:** 2016-10-24

**Authors:** R. Barrois, Th. Gregory, L. Oudre, Th. Moreau, Ch. Truong, A. Aram Pulini, A. Vienne, Ch. Labourdette, N. Vayatis, S. Buffat, A. Yelnik, C. de Waele, S. Laporte, P. P. Vidal, D. Ricard

**Affiliations:** 1 Cognition and Action Group, Cognac-G, CNRS, Université Paris Descartes, SSA, Paris, France; 2 Service de chirurgie orthopédique et traumatologie, HEGP, université Paris Descartes, Paris, France; 3 Institut Galilée, Université Paris 13, Villetaneuse, France; 4 Centre des Mathématiques et de Leurs Applications, Ecole Normale Supérieure de Cachan, Cachan, France; 5 Institut de Recherche Biomédicale des Armées, Brétigny-sur-Orge, France; 6 PRM Department, GH St Louis Lariboisière F. Widal, AP-HP, Diderot University, Paris, France; 7 LBM/Institut de Biomécanique Humaine Georges Charpak, Arts et Métiers Paris Tech, 151 Boulevard de l’Hôpital, 75003, Paris, France; 8 Service de Neurologie, Hôpital d’Instruction des Armées de Percy, Service de Santé des Armées, Clamart, France; Northwestern University, UNITED STATES

## Abstract

For diagnosis and follow up, it is important to be able to quantify limp in an objective, and precise way adapted to daily clinical consultation. The purpose of this exploratory study was to determine if an inertial sensor-based method could provide simple features that correlate with the severity of lower limb osteoarthritis evaluated by the WOMAC index without the use of step detection in the signal processing. Forty-eight patients with lower limb osteoarthritis formed two severity groups separated by the median of the WOMAC index (G1, G2). Twelve asymptomatic age-matched control subjects formed the control group (G0). Subjects were asked to walk straight 10 meters forward and 10 meters back at self-selected walking speeds with inertial measurement units (IMU) (3-D accelerometers, 3-D gyroscopes and 3-D magnetometers) attached on the head, the lower back (L3-L4) and both feet. Sixty parameters corresponding to the mean and the root mean square (RMS) of the recorded signals on the various sensors (head, lower back and feet), in the various axes, in the various frames were computed. Parameters were defined as discriminating when they showed statistical differences between the three groups. In total, four parameters were found discriminating: mean and RMS of the norm of the acceleration in the horizontal plane for contralateral and ipsilateral foot in the doctor’s office frame. No discriminating parameter was found on the head or the lower back. No discriminating parameter was found in the sensor linked frames. This study showed that two IMUs placed on both feet and a step detection free signal processing method could be an objective and quantitative complement to the clinical examination of the physician in everyday practice. Our method provides new automatically computed parameters that could be used for the comprehension of lower limb osteoarthritis. It may not only be used in medical consultation to score patients but also to monitor the evolution of their clinical syndrome during and after rehabilitation. Finally, it paves the way for the quantification of gait in other fields such as neurology and for monitoring the gait at a patient’s home.

## Introduction

Gait analysis plays an important role in the study of lower limb osteoarthritis on two grounds: first, osteoarthritis has important repercussions on gait biomechanics [1–4]. It rapidly worsen the prognosis for the affected joints, and on the long term affect the intact ones, which further compromises the mobility of the patients. Second, the functional syndrome, *ie* the limp evaluated with infrared markers, is well correlated with the severity of the pathology [5]. By using stereophotogrammetry and force plates in gait laboratories, compared to matched controls, knee osteoarthritis patients had reductions in walking speed [6–8], lower cadence [9,10], longer double support time [9,11] and a smaller stride length [12]. That is, gait analysis would be useful to quantify precisely the severity of osteoarthritis in a given patient. However, until recently, gait laboratories were too expensive and complex to be utilized in daily practice. This explains that clinical scores remains the gold standard to evaluate the severity of the pathology up to these days [13–15]. The Western Ontario and MACmaster Universities osteoarthritis index (WOMAC) is actually the most largely used of these scores in rheumatology for lower limb osteoarthritis to assess pain, stiffness, and physical function in patients. WOMAC is considered to be reliable, sensitive and adapted to clinical practice [16–18] and therefore, it is used in most osteoarthritis clinical studies [19,20]. It remains that clinical scores are inherently subjective, as they are based on the patient’s verbal reports and on the clinician’s visual skills and interpretations. For instance, the WOMAC index does not accurately reflect walking performances [21,22] and clinical scores have a lack of sensitivity for identifying changes of balance and walking in mild to moderate disease severity [23].

In that context, skin-mounted accelerometers seem to be well-suited for investigating gait kinematics in osteoarthritis patients [24]. They are inexpensive and non-invasive devices and, more importantly, they are suited for routine clinical practice. In particular, they can be used to evaluate gait using a standard protocol, which involves walking ten meters forward and ten meters back on a level surface at a self-selected walking speed [25–31]. An essential point using gait analysis in the everyday consultation is to extract from the raw data, automatically and in real time, useful parameters for the clinician. To begin, step detection and gait cycle identification are critical for computing gait parameters. By hand, it is time consuming and unfit for clinical practice [3,13,32,33]. On the other hand, the automated routines available for step detection are not robust because they are based on *a priori* predetermined threshold values [34,35]. In addition, step detection automated routines are based on the assumption that steps have stable kinematics, which is not the case in pathological conditions [34,36–40].

Inertial sensors are suitable for quantifying gait performance directly at the routine consultation level. For this use, the quantification can be driven by real-time and low-powered software. Advanced trunk accelerometric parameters have been found useful for detecting pathological gait [41]. Nevertheless, complex gait parameters often require previous step detection, which requires extensive and time-consuming computation for sufficient robustness. As well, the clinical meaning of complex gait parameters is not always clear, although recent papers have made substantial efforts to clarify this point [42]. Still, this situation is unfortunate because straight-forward gait parameters (mean or root mean square [RMS]) for the signals often reveal clinically interpretable results [41,43]. Therefore, simple parameters such as the RMS remain commonly used but often only for the lower back sensor [28,41,44–48]. They often show differences between the pathological and healthy gait. These simple parameters have not been explored at other key anatomical landmarks of the body.

Finally, the gait parameters have widely been developed in complex neurological limping models such as Parkinson disease, cerebral palsy or peripheral neuropathy and not in osteoarthritis, in which pain is believed to be the major limping cause and for which the simple gait parameters could have a direct, understandable clinical meaning [41].

Hence, we have tried to revisit the problem of gait analysis in osteoarthritis patients in daily practice using four inertial motion units (IMU) strapped to the head, lower back (L3-L4) and feet. We have also designed a new automated and online method of gait analysis. This method was then evaluated by comparing its outcome to the severity of the lower limb osteoarthritis evaluated with the WOMAC index in a cohort of 48 patients and 12 control subjects.

## Methods

### Subjects

All subjects (patients and control subjects) were coming for a clinical consultation at the orthopedic surgeon’s office (ThG) during three consecutive months. All consecutive patients or control subjects reaching the inclusion criteria during the inclusion period were included in the study.

All patients had hip or knee osteoarthritis diagnosed by an orthopedic surgeon (ThG) and graded with the WOMAC index (0 to 96). Patients had neither vestibular, neurological, or musculoskeletal disorders, nor any fractures of the lower extremity, nor rheumatoid arthritis or generalized osteoarthritis. Forty-eight patients with lower limb osteoarthritis were included (43 to 90 years, mean 70.9 years). Patients were divided into 2 severity groups of equal size separated by the median of the WOMAC index: the moderately impaired group (G 1) and the severely impaired group (G 2). The median value of the WOMAC index was 45/96. This median-based repartition was chosen in order to maximize the power of the statistical analysis.

The control subjects had no orthopedic nor neurological problem that could affect their gait pattern. Twelve control subjects were included (40 to 87 years, mean 60.8). They formed the age-matched control group (G 0). The mean and standard deviation (SD) of the age, body mass index (BMI) and WOMAC index of each group are shown in Table 1.

**Table 1:** Age body mass index (BMI) and WOMAC index mean (upper case) and standard deviation (lower case) of group 1 and group 2 patients with symptomatic lower limb osteoarthritis and age matched controls.

Group	Number	Age	BMI	WOMAC
**0**	12	63,2	25,2	0,0
		17,1	4,6	0,0
**1**	24	70.5	26.8	14,1
		9.5	5,7	10,0
**2**	24	70,5	28,2	62,58
		14,9	5,5	14,0

To assess the test–retest validity of the discriminating parameters, we checked their variability with IMU placement. For the sensor-placement control experiment 1, 2 healthy controls (age 22 and 23 years) performed 5 walking trials with sensors placed by 2 different operators at each trial. For the sensor-placement control experiment 2, these 2 subjects also performed 9 walking trials with displacement of the sensor along the antero-posterior (AP) axis and the medio-lateral (ML) axis in terms of the reference position (from -20 to +20 mm in 5-mm increments). Coefficients of variation ($CV=\frac{\mu }{\sigma }$) were evaluated for these 2 experiments, where μ is the mean and *σ* the standard deviation of the parameters over all trials for each sensor control experiment. A CV < 5% was considered correct and < 10% acceptable.

The study was validated by a local ethic comity (Comité de Protection des Personnes Ile de France II, n°CPP 2014-10-04 RNI) and both patients and control subjects gave their written consent to participate.

### Instrumentation

Linear accelerations and angular velocities of the head, lower back (L4-L5 vertebra) and feet were collected using four IMUs including triaxial accelerometers, gyroscopes and magnetometers (XSens®, Culver City, CA, USA, MTw Measurement Units, 3,5h LiPo battery, 27g, 3,5x5,8x1,0cm^3, +/-16g, +/-1200deg/s, 100Hz, errors 0,003m/s^2^ and 0,05deg/s), fixed with manufacturer-designed adhesive straps and connected through WiFi with a computer.

### Defining the sensor linked frame and the doctor’s office linked frame

The accelerations and the angular velocities of the four IMUs can be expressed in the sensor linked frame and in the doctor’s office linked frame.

The IMUs were fixed and aligned with respect to the body in the following way. The head sensor was positioned on the center of the forehead. The antero-posterior (AP) axis of the frame linked to the head sensor was the normal to the forehead surface. The medio-lateral (ML) axis was set parallel to the line joining the left temple and the right temple. The vertical (V) axis completed the orthonormal frame. The lumbar sensor was positioned at L4-L5 level. The AP axis of the frame linked to the lumbar sensor was normal to the back surface. The ML axis was set parallel to the line joining the right anterior superior iliac spine and left anterior superior iliac spine. The V axis completed the orthonormal frame. Each foot sensor was positioned at the center of the dorsal face of each foot. The V axes of each frame linked to each foot sensor were the normals to the dorsal surfaces of each foot. The AP axis was set parallel to the longitudinal direction of the foot. The ML axis completed the orthonormal frame. Positive directions for the axes were not defined because all computed gait parameters are independent of this orientation.

The doctor’s office frame was the fix frame linked to the doctor’s office. The V axis of the doctor’s office linked frame was aligned with the gravity. The horizontal plane (H) was the plane normal to the V axis. AP and ML axes were not defined in the doctor’s office linked frame. The change of frame from the sensor linked frames to the doctor’s office linked frame was done with an algorithm [49,50] based on the XSens® 3D magnetometer measurement. We used the manufacturer’s rotation matrix as described and validated by Cognolato [50].

### Experimental design and data acquisition

The WOMAC index was evaluated and recorded by the same experimented orthopedic surgeon (ThG). The questions were always asked in the same order with the validated text. After the sensor fixation, the participant was instructed to execute the following steps: stand quiet for six seconds, walk ten meters at a preferred walking speed, make a U-turn, walk the ten meters back and stand quiet for two seconds.

Participants could keep their clothes and their shoes on. Participants with high heels (>2 cm) were asked to do the exercise without their shoes. Each participant completed two trials of this exercise to improve the reliability of the measure.

### Data processing

**Fig 1:**
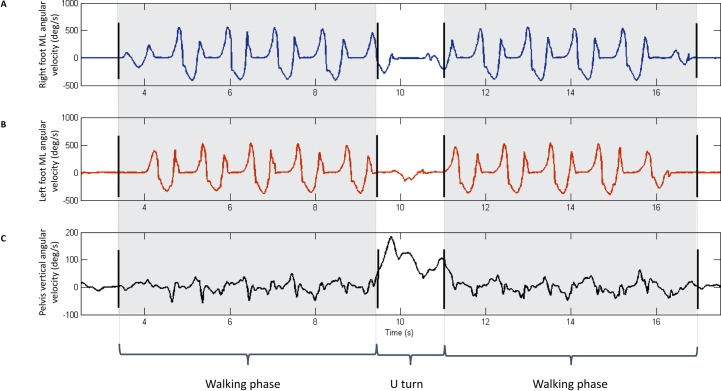
Representative data and manual phase annotation result for one healthy participant performing a 10 meters go and 10 meters back walking exercise at self-selected walking speed. Black bars stand for manual annotation. Dashed zone corresponds to the walking phases. The walking parts of the signal were taken for parameter computation. (**A**)–Representative ML lateral angular velocity in the sensor linked frame for right foot. (**B**)—Representative ML lateral angular velocity in the sensor linked frame for left foot. (**C**)–Representative V angular velocity in the sensor linked frame for L3-L4.

Each phase of the exercise (quiet standing, walking and U-turn) was manually annotated without any step detection (RB). All parameters were computed on the concatenated signal of the walk phases of the exercise (Fig 1). One given parameter *p* is defined by a *sensor = {head*, *lower back*, *ipsilateral foot*, *contralateral foot}*, a *frame = {sensor*, *office}*, an *axis = {AP*,*ML*,*V}* if the frame is the sensor-linked frame or an *axis = {H*, *V}* if the frame is the doctor’s office-linked frame (H for horizontal plane), a signal *sig = {acceleration*, *angular velocity}* and a statistical tool *stat = {mean*, *RMS}*. Thus we computed the following:

$$p_{sensor,frame,axis,sig,mean}=mean|{sig}_{sensor,frame,axis}|$$

$$p_{sensor,frame,axis,sig,RMS}=RMS|{sig}_{sensor,frame,axis}|$$

where |.| is the absolute value and where in the case of n values *x* = {*x*_1_,*x*_2_,…,*x*_*n*_}:

- the mean is defined by $mean(x)=\frac{1}{n}(x_{1}+x_{2}+\cdots +x_{n})$
- the RMS is defined by $RMS(x)=\sqrt{\frac{1}{n}(x_{1}^{2}+x_{2}^{2}+\cdots +x_{n}^{2})}$

For each parameter, the mean of the two trials was taken. Sixty parameters were computed, fifteen for each sensor (Table 2).

**Table 2:** Acceleration and angular velocity parameters in the sensor linked frames and the doctor’s office linked frame. RMS for root mean square.

**Sensor linked frame**		
**Axis and plane**	**Acceleration**	**Angular velocity**
Meadial lateral (ML)	-	Mean
	RMS	RMS
Anterior posterior (AP)	-	Mean
	RMS	RMS
Vertical (V)	-	Mean
	RMS	RMS
**Doctor’s office linked frame**		
**Axis and plane**	**Acceleration**	**Angular velocity**
Horizontal (H) plane	Mean	-
	RMS	-
Vertical (V) axis	Mean	Mean
	RMS	RMS

The parameters were also computed by sliding the manually-annotated computation window one second earlier and one second later to take the error of the manual phase annotation into consideration (see Results section). The parameters affected by the gravity component were not studied because they were too sensor’s positioning dependent. These parameters were: the mean of the norm of the acceleration in the sensor linked frames in the AP, ML and V directions on the four markers *i*.*e*.:

$$p_{\{head,lower\;back,feet\},sensor,\{AP,ML,V\},acceleration,mean}$$

Gravity component of the acceleration was not removed. The angular velocities in the horizontal plane in the doctor’s office frame was not studied because of the absence of clinical meaning of this parameter *i*.*e*.:

$$p_{\left\{head,lower\;back,feet\},office,H,angular\;velocity,\{mean,RMS\right\}}$$

Mean walking velocity was computed by dividing the walking distance (20 m) by the duration of the walking phases.

### Statistical analysis

A one-way analysis of variance (ANOVA) with Tukey pairwise comparison test and a one-way analysis of covariance (ANCOVA) with age and BMI as covariate with Tukey pairwise comparison were performed on all three groups on all the 61 parameters. Mean walking velocity was not taken as covariate because it is known to decrease with lower limb osteoarthritis severity [25]. We defined a discriminating parameter as a parameter that showed statistical differences using an ANOVA analysis with a Tukey pairwise comparison test (p-value set under 0, 05) between all three groups (G1vsG2, G2vsG3 and G1vsG3).

## Results

### Data processing

We could manually annotate the initial quiet-standing phase, the go-walking phase, the U-turn and the back-walking phase for all 48 lower limb osteoarthritis patients and the 12 control subjects. Representative data and manual phase annotation results for one control subject performing a 10 meters forward and 10 meters back walking task at a self-selected walking speed are shown in Fig 1. The cumulative error for the manual exercise phase annotation was 1 second. The relative errors due to the manual annotation error on the parameters were 5% on average for the mean of the acceleration in the horizontal plane on the ipsilateral foot. The errors did not change the statistical significance of the in-between group differences shown by the discriminating parameters.

### Parameters and statistical analysis

**Fig 2:**
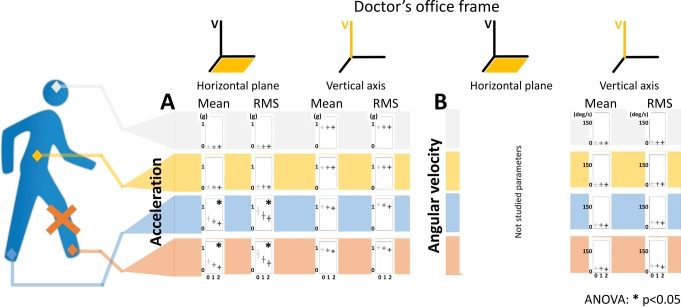
Selected 24 parameters out of the 60 IMU based parameters computed in the doctor’s office linked frame obtained from 4 IMUs on 12 control subjects and 48 patients during a 10 meters go and 10 meters back walking task. Sensor location are shown on the walking silhouette by colored diamonds: grey for the head, yellow for the sacrum, blue for the contralateral foot and red for the ipsilateral foot. The red cross of the walking silhouette indicates the ipsilateral foot to the lesion defined by the side where the patient is the more symptomatic. Each parameter is represented by a bar diagram. The row indicate the location of the sensor and whether the parameters is computed on an acceleration (**A**) or an angular velocity signal (**B**). The columns indicate whether the parameter is computed on the horizontal plane or on the vertical axis and whether the parameter is a mean or a RMS of the norm of the walking signal. In each bar diagram, the parameter is represented as a function of the severity. The results are shown by a modulated grey cross: horizontal bar stands for mean and vertical bar stands for the standard deviation. Light grey represents the healthy group (G0), medium grey the moderately impaired group (G1) and dark grey the severely impaired group (G2). The parameters marked by a star (*) are the discriminating parameters (parameters that show significant difference between the three WOMAC index defined severity groups). The statistical analysis was performed with an ANOVA analysis and a Tukey pairwise comparison test (p-value set at 0.05). RMS stands for root mean square and V for vertical axis.

Looking at the 60 IMU-based parameters we found (S1 Table):

- in the sensor linked frames: no discriminating parameters (results not shown).
- in the doctor’s office linked frame: the mean and the RMS of the norm of the acceleration in the horizontal plane for the contralateral (p-values respectively G0vsG1 = 0.011; G1vsG2 = 0.013; G0vsG2<0.0001 for mean and G0vsG1 = 0.010 G1vsG2 = 0.026; G0vsG2<0.0001 for RMS) and the ipsilateral (p-values respectively G0vsG1 = 0.002; G1vsG2 = 0.0004; G0vsG2<0.0001 for mean and; G0vsG1 = 0.001; G1vsG2 = 0.001; G2vsG0<0.0001 for RMS) foot were discriminating parameters (Fig 2). In our predefined formalism these parameters are *p*_{*ipsilateral foot*,*controlateral foot*},*office*,*H*,*acceleration*,{*mean*,*RMS*}_.

- These parameters can be

In the sensor linked frame, angular velocities around the ML axis on the ipsilateral and contralateral feet didn’t appear to be discriminating parameters, but showed statistical significant differences between the group of control subjects and the two groups of patients (results not shown).

No parameters from the lower back and no parameter from the head were discriminating parameters.

We found that the mean and RMS of the norm of the acceleration in the horizontal plane in the doctor’s office linked frame for contralateral and ipsilateral feet still met our definition of discriminating parameters with age and BMI as covariate.

**Fig 3:**
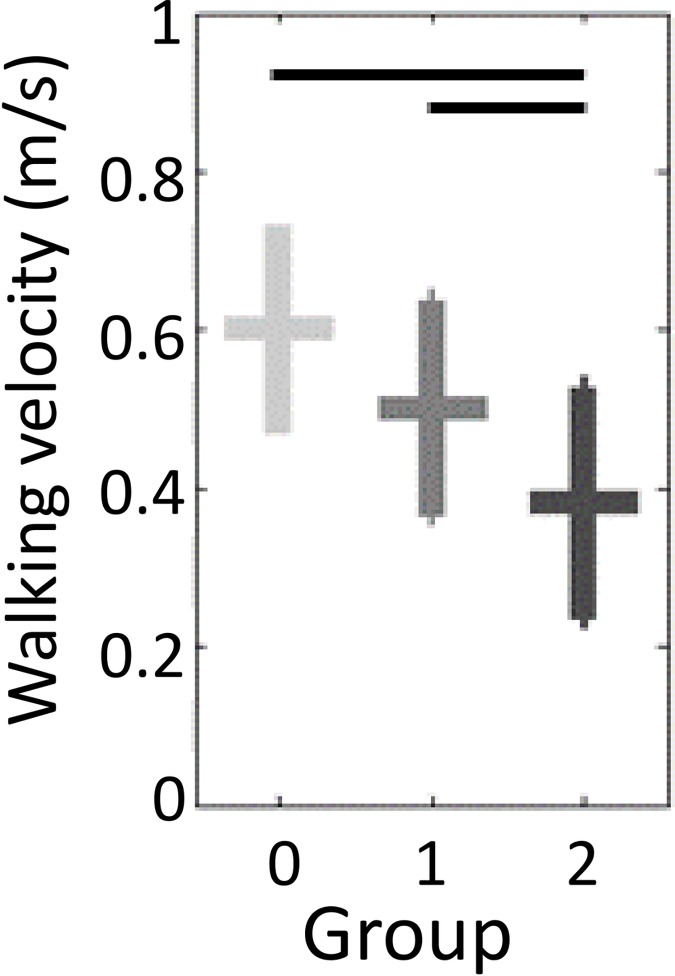
Mean walking velocity as a function of the WOMAC index based osteoarthritis severity groups. The results are shown by a modulated grey cross: horizontal bar stands for mean and vertical bar stands for the standard deviation. Light grey represents the healthy participants, medium grey the moderately impaired group and dark grey the severely impaired group. Black horizontal bars show the statistical differences between the groups computed with an ANOVA analysis and a Tukey pairwise comparison test (p-value set at 0.05).

For walking velocity, differences were significant between G0 and G2, G1 and G2. No significant difference in walking velocity was found between G0 and G1 (Fig 3). Thus, walking velocity was not a discriminating parameter.

Sensor-placement control experiment 1 gave a CV < 5% and experiment 2 a CV < 10% for the mean of the norm of the acceleration in the horizontal plane and the RMS of the norm of the acceleration in the horizontal plane (Table 3).

**Table 3:** Sensor-placement control experiment 1 (Exp. 1): coefficient of variation (CV; mean/SD) of the mean and root mean square (RMS) of the norm for acceleration in the horizontal plane in the right foot for 2 subjects over 5 walking trials with renewal of the sensor placement at each trial. Sensor-placement control experiment 2 (Exp. 2): CV over 9 walking trials (-20; -15; -10; -5; 0; 5; 10; 15; 20 mm) with displacement of the sensor in increments of 5 mm along the antero-posterior axis and medio-lateral axis in terms of the reference position. Values are in percentages.

		Mean	RMS
**Exp. 1**	**Subject 1**	3.5*	4.3*
	**Subject 2**	0.9*	2.1*
**Exp. 2**	**Subject 1**	7.4	8.9
	**Subject 2**	2.9*	4.0*

* CV < 5%.

## Discussion

The correlation between lower limb osteoarthritis severity and stereophotogrammetry is well established [5,26,27,51–57]. In contrast, only two studies retrieved the same correlation using inertial sensors [52,54]. We confirm that result here. In addition, to the best of our knowledge, it is the first lower limb osteoarthritis study where the IMU-based gait parameters were extracted without step detection, which is important for daily clinical use. Finally, our results suggest that two IMUs placed on the feet are sufficient to quantify the severity of inferior limb osteoarthritis, which further improves the use of the method in daily practice.

We compared 48 patients and 12 control subjects walking 10 meters forward and 10 meters back under clinical consultation conditions. The four-IMUs-based method showed a discrimination capacity of clinical severity groups for 4 of the 60 parameters tested. These discriminating parameters were: mean and RMS of the norm of the acceleration in the horizontal plane in the doctor’s office linked frame for the contralateral and the ipsilateral feet. The results remained statistically significant with BMI and age as covariate. The absence of clinical correlation with parameters in the head and lower back reflected that lower limb osteoarthritis impacted the kinematics of the painful segment more than the upper body, which, to the best of our knowledge, has not been specifically shown previously [1,12,56,58–61]. However, it cannot be excluded that a more precise method of measurement, such as stereophotogrammetry, could reveal subtle differences. It remains that one important conclusion would be that two sensors placed at the feet, would be sufficient in daily practice to rate osteoarthritis severity.

Walking speed is known to influence gait parameters [62] and osteoarthritis reduces walking speed. Hence, the question is whether the influence of osteoarthritis severity on the gait parameters was solely caused by the reduction of walking speed, or if osteoarthritis *per se* led to a change of gait pattern. To analyze the change of walking pattern independently from the walking speed, a first method is to walk at a predetermined walking speed [1,3,12,33,56,58,63–65]. It requires dedicated material (treadmill), which is not suited in daily clinical practice and it does not allow to capture natural and repeatable walking patterns [25]. A second method is to select subgroups of participants walking at their preferred walking speed matched in walking speed [66]. But, the subgroups do not reflect the general populations of the whole severity groups [25]. A third method would be to set walking speed as covariate [63,67,68]. As walking speed is inherently linked to disease progress, and its mean value tends to decrease with increasing levels of disease severity, this technique is inappropriate [25]. Therefore, we chose to have participant walking at preferred walking speeds. Using that method, we showed on our dataset that walking velocity was not a discriminating parameter when comparing G0 and G1. Altogether, this negative result suggests that osteoarthritis *per se* caused a change of gait pattern, independent from the walking velocity. Pain could likely be a factor.

Our method gave a global view of the gait kinematics, which summed up the impacts of ostheoarthritis at the hip, knee and ankle joints levels. Also, ipsilateral and contralateral sides were defined with respect to the more symptomatic side of the patient. Therefore, our approach may help to objectively rate lower limb osteoarthritis severity in daily clinical practice but it is not suited to gain a detailed insight in the walking pattern of these patients [52].

The manual phase annotation of the walking exercise we used saved time but could have lowered the robustness of our method. However, we showed that the errors due to manual annotation didn’t change the statistical validity of the discriminating parameters in our study. Computation of gait parameters in the sensor-linked frame is prone to lower the reproducibility of the parameters because it is biased by the inherent variability of the positions of the sensors [44,69]. This explains why in our study, robust discriminating accelerometric parameters for lower limb osteoarthritis severity were all found in the doctor’s office linked frame.

Two aspects of the positioning of the sensors may affect gait parameters by using IMUs: the orientation and position of the sensor on the measured body segment [70–73]. In the present study, all discriminating parameters were computed from the laboratory frame (*i*.*e*. the frame in which the vertical axis and horizontal plane are independent of the initial orientation of the sensor). Nevertheless, with the effect of the position of the sensor on the body segment, the CV was < 5% for our discriminating parameters, for realistic placement errors (we estimated our error as routine to be about 10 mm), and < 10% for extreme placement errors. Indeed, special care is needed for placement of the sensor, but this positioning had moderate impact on the parameters we propose.

We compared the IMU-based gait parameters and lower limb osteoarthritis assessed by the WOMAC index, which is a purely clinical score. Classically, inertial sensor based studies use the Kellgren and Lawrence radiographic score to rate knee osteoarthritis [5,26,27,53–57]. Radiographic knee osteoarthritis severity is known to have poor correlation with the clinic namely gait disturbance [74,75]. Radiographic osteoarthritis can be clinically silent [26], which could explain the inconsistent correlation between gait analysis and radiographic-based lower limb osteoarthritis severity [59]. Again, it can be hypothesized that pain commands walking strategies.

Finally, beyond the fact that we designed an automated method of gait quantification, adapted to daily practice, our results gave some insight in the impact of lower limb osteoarthritis on locomotion. The most relevant results of our study are the decrease of the mean and RMS of norm of the acceleration in the horizontal plane on both feet with disease severity. It could result from a diminution of movement in the AP direction due to pain. This interpretation had been suggested in studies relying on local peak amplitudes [13,54,76,77]. Liikavainio *et al*. (2010) have also hypothesized that patients use a different strategy to brake the forward movement of the swinging leg before floor contact. This strategy could explain both the reduction of our global parameters and the increase of the local peaks in patients reported by others.

## Conclusion

Our study showed that by using two IMUs placed on both feet and a signal processing method without step detection, we could objectively quantify limp in lower-limb osteoarthritis. This finding underlines the importance of measuring key anatomical landmarks and accessible gait parameters in exploring limp by using IMUs and severity grading. Although the proposed method still had some limitations, it provided new, automatically computed parameters that could be used for the comprehension of lower limb osteoarthritis in current medical practice. It may not only be used in medical consultation to score patients, but also to monitor the evolution of their clinical syndrome during and after rehabilitation. Finally, it paves the way for the quantification of gait in other fields such as neurology and for home monitoring.

## Supporting Information

S1 Table Lower limb osteoarthritis severity group, WOMAC score, BMI, age, walking velocity and the 60 parameters for the 12 control subjects and the 48 patients. Each parameter is defined by: a *sensor = {head*, *lower back*, *ipsilateral foot*, *contralateral foot}*; a *frame = {sensor*, *office}*; an *axis = {AP*,*ML*,*V}* if the frame is the sensor-linked frame or an *axis = {H*, *V}* if the frame is the doctor’s office-linked frame (H for horizontal plane); a signal *sig = {acceleration*, *angular velocity}* and a statistical tool *stat = {mean*, *RMS}*. The parameter *ipsilateral foot-office-H-acceleration-mean-modified* corresponds to the parameter *ipsilateral foot-office-H-acceleration-mean* computed with the cumulative error for the manual exercise phase annotation that was estimated at 1 second. Accelerations are given in g and angular velocities in deg/s. AP antero-posterior, ML medio-lateral, V vertical, H horizontal plane, RMS root mean square, BMI body mass index.
(XLSX)
